# Supplementary material for: From sequence to enzyme mechanism using multi-label machine learning
Source: BMC Bioinformatics. 2014 May 19;15:150. doi: 10.1186/1471-2105-15-150 (PMC4229970; doi:10.1186/1471-2105-15-150)
Supplement: Additional file 2 — Java code of ml2db. Additional file ml2db_code.tar.gz contains the Java source code to run the multi-label machine learning experiments and save the results to database. The code’s Javadoc is included. [file 1471-2105-15-150-S2.zip › additional file 2/ml2db/ml2db/doc/mulan/classifier/package-summary.html]

mulan.classifier


JavaScript is disabled on your browser.


- Overview
- Package
- Class
- Use
- Tree
- Deprecated
- Index
- Help

- Prev Package
- Next Package

- Frames
- No Frames

- All Classes

# Package mulan.classifier

- Interface Summary

  | Interface | Description |
  | --- | --- |
  |  |  |
  | --- | --- |
  | MultiLabelLearner | Common root interface for all multi-label learner types. |
- Class Summary

  | Class | Description |
  | --- | --- |
  |  |  |
  | --- | --- |
  | MultiLabelLearnerBase | Common root base class for all multi-label learner types. |
  | MultiLabelOutput | Class representing the output of a `MultiLabelLearner`. |
- Exception Summary

  | Exception | Description |
  | --- | --- |
  |  |  |
  | --- | --- |
  | InvalidDataException | Exception thrown by the `MultiLabelLearner` when presented with invalid data or data which can not be processed by the specific learner type. |
  | LearnerException | Represents a root base class for exceptions thrown by learners. |
  | ModelInitializationException | Exception thrown by the `MultiLabelLearner` when learner is queried for prediction on data before model is internally built by learning from training data. |

- Overview
- Package
- Class
- Use
- Tree
- Deprecated
- Index
- Help

- Prev Package
- Next Package

- Frames
- No Frames

- All Classes
